# Comparative Analysis of Perceval and Conventional Bovine Bioprosthetic Valves in Aortic Valve Replacement: Hemodynamics, Reverse Remodeling, and Long-Term Outcomes

**DOI:** 10.3390/jcm14113899

**Published:** 2025-06-01

**Authors:** Shen-Che Lin, Jer-Shen Chen, Jih-Hsin Huang, Kuan-Ming Chiu, Chih-Yao Chiang

**Affiliations:** 1 Department of Cardiovascular Surgery, Cardiovascular Center, Far Eastern Memorial Hospital, New Taipei City 220216, Taiwan; femh97996@femh.org.tw (S.-C.L.); femh76818@femh.org.tw (J.-S.C.); femh87919@femh.org.tw (J.-H.H.); 2 Department of Electrical Engineering, Yuan Ze University, Taoyuan 320315, Taiwan; 3 Division of Cardiovascular Surgery, Department of Surgery, School of Medicine, National Defense Medical Center, Taipei 114201, Taiwan

**Keywords:** aortic stenosis, aortic valve replacement, Perceval sutureless valve

## Abstract

**Background/Objectives:** Surgical aortic valve replacement effectively relieves left ventricular afterload and promotes reverse remodeling in patients with severe aortic stenosis. The Perceval prosthesis offers a hybrid approach, combining complete annular decalcification with sutureless deployment. This design allows for reduced operative times and potentially larger effective orifice areas. However, comparative data with conventional stented bioprosthetic valves remain limited, particularly regarding reverse remodeling, hemodynamic performance, and long-term clinical outcomes. **Methods**: In this retrospective cohort study, 115 patients underwent aortic valve replacement with either the Perceval valve (*n* = 44) or conventional stented bovine pericardial valves (*n* = 71). **Results:** The Perceval group showed a 100% procedural success rate with no in-hospital mortality, significantly shorter cardiopulmonary bypass and cross-clamp times, larger effective orifice areas, and a lower incidence of patient–prosthesis mismatch. Both groups demonstrated favorable left ventricular mass regression and reverse remodeling. The rates of paravalvular leakage, permanent pacemaker implantation, and redo aortic valve replacement were comparable between groups. Multivariate Cox regression identified the follow-up indexed left ventricular mass as an independent predictor of major adverse cardiac and cerebral events. **Conclusions**: In this study, the Perceval valve was associated with promising hemodynamic characteristics and procedural efficiencies, particularly in cases with small aortic annuli and during minimally invasive procedures. The valve was associated with reverse ventricular remodeling and clinical outcomes that appeared similar to those of conventional stented bioprostheses. These observations suggest it may represent a potential alternative option for surgical aortic valve replacement in appropriate clinical scenarios. However, randomized control trials are needed to confirm these associations.

## 1. Introduction

Aortic stenosis (AS) is the most prevalent valvular heart disease in developed countries, with valvular sclerosis affecting over 25% of individuals aged 65 and older [1,2]. AS primarily results from the progressive, inflammation-mediated calcification of the valve leaflets, leading to reduced leaflet mobility, valvular narrowing, and chronic pressure overload that elevates the left ventricular afterload [3,4]. In individuals with a bicuspid aortic valve, abnormal turbulent flow further accelerates sclerotic degeneration [5]. In response to chronic pressure overload, the left ventricle undergoes adaptive concentric hypertrophy to preserve wall stress homeostasis and prevent functional ischemia [4,6]. Although initially compensatory, this remodeling reduces mechanical efficiency, while progressive hypertrophy increases myocardial stiffness, leading to diastolic dysfunction and ultimately culminating in the ventricular decompensation process to heart failure [7,8,9,10,11].

Aortic root enlargement allows for the implantation of larger prostheses in small aortic roots [12,13,14,15]. Additionally, advances in prosthetic valve materials help minimize the frame profile, enhancing the effective orifice area (EOA), reducing patient–prosthesis mismatch (PPM), optimizing flow dynamics, and improving both the valve performance and durability [16]. Advancements from sutured to sutureless valves reduce the need for multiple sutures, minimizing the morbidity of cardiopulmonary bypass [16,17,18,19]. Transcatheter technologies offer less invasive annular anchoring and avoid ischemia–reperfusion injury [20,21,22]. However, in Transcatheter aortic valve implantation (TAVI), the EOA may be limited by retained native leaflets and annular calcification, increasing the risk of paravalvular leakage and conduction disturbances [23,24].

Perceval valves combine the benefits of surgical aortic valve replacement (AVR) and TAVI by enabling rapid, sutureless deployment with a self-expanding nitinol frame after complete valve excision and annular decalcification [18,19]. This approach reduces the operative time and enhances the EOA, particularly in patients with small aortic annuli. However, concerns persist regarding paravalvular leakage, conduction disturbances, and long-term valve durability. Comparative data between Perceval and conventional stented bovine valves remain limited, particularly in relation to hemodynamics, ventricular remodeling, and clinical outcomes. This study compares surgical aortic valve replacement (AVR) using the Perceval valve with that using conventional valves, focusing on the primary endpoints of procedural safety, mortality, and hospital or late adverse events, and the secondary endpoints of the hemodynamic performance and ventricular reverse remodeling.

## 2. Materials and Methods

### 2.1. Study Design and Population

This single-center retrospective case–control study analyzed 269 patients who underwent surgical AVR from January 2018 to December 2023. Inclusion criteria were severe symptomatic AS and elective AVR with a bovine pericardial prosthesis, with or without concomitant coronary artery bypass grafting (CABG). Exclusion criteria included moderate to severe aortic regurgitation, multivalve disease, aortic pathology (e.g., dissection, dilation), and prior cardiac surgery. Among the 269 patients, 115 met the inclusion and exclusion criteria. Surgical approaches included median sternotomy or minimally invasive cardiac surgery, based on surgeon preference. Prosthetic type was determined through shared decision making between the surgeon and patient, primarily guided by clinical considerations and financial factors. Patients were grouped by prosthetic type: 44 received sutureless Perceval valves and 71 received conventional stented bovine valves. Clinical, operative, echocardiographic, and late outcomes were analyzed by perioperative course, hemodynamic performance, and late events. This study was approved by the Institutional Review Board of Far Eastern Memorial Hospital (111256-E).

### 2.2. Data Collection and Measurement

Baseline data included age, sex, body surface area (BSA), comorbidities, atrial fibrillation, ventricular dysfunction, morphology of aortic annulus and bicuspid valve, remodel mode, and echocardiographic parameters. Operative variables comprised surgical approach, prosthesis size, concomitant procedures, cardiopulmonary bypass (CPB) duration, and aortic cross-clamp time. In-hospital and late events included conduction disturbances, paravalvular leakage, low cardiac output syndrome, major adverse cardiac and cerebral events (MACEs), reoperation, and mortality. Follow-up data were collected through outpatient medical records and transthoracic echocardiography. Electrocardiographic evaluation included assessment of cardiac rhythm, QRS duration, bundle branch blocks (including new onset left bundle branch block, LBBB), and atrioventricular (AV) block. Conduction disturbances were defined as new-onset LBBB with a QRS duration >120 ms or PR interval prolongation associated with third-degree AV block or advanced second-degree AV block and clinically significant postoperative symptoms. Permanent pacemaker implantation (PPI) was indicated in cases of conduction disturbances accompanied by symptomatic bradyarrhythmia or hemodynamic instability. Patients with pre-existing permanent pacemaker implantation prior to the intervention were excluded from the analysis. Accordingly, they were not included in the time-to-event comparisons between the two groups. Transthoracic echocardiography was performed preoperatively, during hospitalization, and at 6–12 months postoperatively using a Philips iE33 system with 2.5–3.5 MHz transducers. Hemodynamic parameters included mean and peak transvalvular gradients, EOA, and severity of aortic stenosis according to standard guidelines. Left ventricular (LV) geometry was assessed by M-mode, including the LV end-systolic dimension (LVESD) and LV end-diastolic dimension (LVEDD), interventricular septal thickness (IVST), and posterior wall thickness (PWT). LV volumes, LV end-systolic volume (LVESV), and LV end-diastolic volume (LVEDV) were calculated using Simpson’s method. Stroke volume (SV) was derived as left ventricular outflow tract (LVOT) area × velocity time integral (VTI) and indexed by body surface area (BSA). EOA was calculated using the continuity equation $EOA=\frac{{Area}_{LVOT}\times {VTI}_{LVOT}}{{VTI}_{AV}}$, $indexed\;EOA=\frac{EOA}{BSA}$. Postoperative EOA was adapted from projected values in prior studies and indexed to body surface area (EOAI) to assess PPM [8,10]. PPM was classified as severe (EOAI ≤ 0.65 cm^2^/m^2^), moderate (0.65–0.85 cm^2^/m^2^), or absent (>0.85 cm^2^/m^2^). The velocity–time integral (VTI) ratio is the dimensionless index for the assessment of AS severity. VTI ratio $=\frac{LVOT\;VTI}{Aortic\;valve\;VTI}$; LV mass (LVM) was calculated using the ASE formula [25]. The equation for end-systolic meridional wall stress was originally derived by Grossman et al. [26,27].

LVM = 0.8 × [1.04 × ((IVSd + LVIDd + PWTd)^3^ − LVIDd^3^)] + 0.6. Indexed LVM $=\frac{LV\;mass}{BSA}$; relative wall thickness (RWT) was calculated as RWT $=\frac{2\times PWT}{LVEDD}$:

$$End\;systolic\;meridional\;wall\;stress=\frac{1.33\times (LV\;end-systolic\;pressure)\times (LVESD)}{4h\times \left(1+\frac{h}{LVESD}\right)}$$

where $h=\frac{IVST+PWT}{2}$; E/e′ ratio $=\frac{Mitral\;inflow\;early\;diastolic\;velocity\;(E)}{Early\;diastolic\;mitral\;annular\;tissue\;velocity\;(e^{\prime })}$.

The E/e′ ratio, derived from mitral inflow (E) and mitral annular tissue (e′) velocities, is a doppler echocardiographic measure of the LV filling pressure. Low cardiac output syndrome was defined as cardiac index less than 2.2 L/min/m^2^ in the presence of clinical signs of hypoperfusion. Pulmonary hypertension is defined as systolic PAP > 35 mm Hg.

### 2.3. Valve Sizing and Implantation Technique

After excising the native valve and completing annular decalcification, valve sizing was performed using manufacturer-specific sizers. All valves were implanted under direct visualization using standard techniques. The Perceval valve (Corcym S.r.l., Milan, Italy), composed of bovine pericardial tissue mounted on a self-expanding nickel–titanium frame coated with Carbofilm™ (Corcym S.r.l., Milan, Italy), was implanted using three guiding sutures placed at the annular nadirs, followed by deployment and balloon dilation to ensure proper expansion and seating [28]. Conventional stented bovine pericardial valves (Mitroflow (LivaNova PLC, London, UK), Perimount (Edwards Lifesciences, Irvine, CA, USA), Magna (Edwards Lifesciences, Irvine, CA, USA), Avalus (Medtronic, Minneapolis, MA, USA) and Inspiris Resilia (Edwards Lifesciens, Irvine, CA, USA)) were implanted using multiple pledgeted sutures around the annulus. Following valve placement, the aortotomy was closed, and patients were weaned from cardiopulmonary bypass after de-airing and transesophageal echocardiographic (TEE) assessment.

### 2.4. Endpoint Definitions and Classification

Primary endpoints included all-cause and cardiac mortality, as well as MACEs [29], defined as a composite of coronary reintervention, stroke, and heart failure-related rehospitalization. Valve-related adverse events such as paravalvular leakage (PVL) [23,28], conduction disturbances [30], valve-related thromboembolism, prosthetic valve endocarditis, and permanent pacemaker implantation were also assessed [31]. Secondary endpoints focused on prosthetic valve function and left ventricular hemodynamic performance, including reverse remodeling and mass regression, evaluated by transthoracic echocardiography. Events were identified during hospitalization and follow-up through comprehensive outpatient medical records.

### 2.5. Statistical Analysis

Paired *t*-tests were used to compare preoperative and follow-up geometric and hemodynamic parameters within groups, while independent *t*-tests assessed differences between subgroups. Categorical variables were analyzed using chi-square or Fisher’s exact tests, as appropriate. Cox proportional hazard models were used to assess associations between clinical and echocardiographic parameters and the risk of MACEs. Due to the small sample size (n = 115) and the limited number of events (31 patients with MACEs), the above regression models were adjusted for age, sex, cardiovascular risk factors, including hypertension, systolic dysfunction (preoperative LVEF < 50%), and dilated LVEDD (>55 mm). Variables were screened via univariate Cox regression (e.g., *p* < 0.10), using stepwise selection, and then a multivariate Cox model was built for adjusted risk analysis. All statistical tests were two-tailed, with significance set at *p* < 0.05, using MedCalc software, version 23.2.1, 2025 (MedCalc Software Ltd., Ostend, Belgium).

## 3. Results

### 3.1. Baseline Demographic and Clinical Characteristics

As shown in Table 1, a total of 115 patients underwent AVR with or without concomitant procedures. The mean age was 65 ± 10 years, and 55% were male. The median follow-up duration was 40 months (95% CI: 35 to 53). Dyslipidemia was the most common comorbidity (74%), followed by hypertension (63%), coronary artery disease (36%), and diabetes (34%). Chronic kidney disease was present in 25% of patients, with 7% requiring hemodialysis. Prior stroke and infective endocarditis were reported in 8% and 3%, respectively. Preoperative conduction disturbances included left bundle branch block (15%), atrial fibrillation (10%), and prior pacemaker implantation (3%). Bicuspid aortic valve morphology was observed in 47% of patients, and 53% had an annular diameter ≤ 21 mm (mean 21 ± 2 mm). At baseline, 76% of patients exhibited concentric hypertrophy, consistent with the pressure-overload response typically seen in AS. Notably, both men and women showed high rates of concentric hypertrophy but different prevalences between genders (men: 70%; women: 83%). However, eccentric hypertrophy was more prevalent in men (men: 18% vs. women: 6%), reflecting gender-related differences in the LV adaptation to chronic pressure overload. Left ventricular dysfunction (LVEF <55%) was present in 15% and systolic pulmonary artery pressure > 35 mm Hg in 22%. Baseline characteristics were comparable between the valve subgroups, except for a higher prevalence of atrial fibrillation in the conventional valve group.

### 3.2. Operative Characteristics and In-Hospital Adverse Events

The details of the implanted aortic prostheses are summarized as follows: A total of 115 patients underwent AVR, with 44 receiving the Perceval valve and 71 receiving conventional stented bioprosthetics. In the Perceval group, the valve sizes included small (n = 13), medium (n = 18), large (n = 10), and extra-large (n = 3). In the conventional group, Mitroflow valves were used in 27 patients (sizes 19:1, 21:14, 23:11, 25:1), Perimount in 17 (21:5, 23:3, 25:6, 27:3), Magna in 15 (21:5, 23:5, 25:5), Avalus in 10 (21:5, 23:3, 25:2), and Inspiris Resilia in 2 (21:1, 23:1). As shown in Table 2, ministernotomy was the most common surgical approach (77%), and 5% of patients had prior AVR. Concomitant procedures included coronary artery bypass grafting (CABG) in 16% and pulmonary vein ablation in 6% of patients. Aortic root augmentation for the small annulus was performed in six patients, all within the conventional valve group. There were no significant differences between the groups in terms of the surgical approach or concomitant procedures. The Perceval group demonstrated a 100% procedural success rate with no in-hospital mortality. Additionally, the cardiopulmonary bypass time was significantly shorter in the Perceval group [93 min (95% CI: 88–102)] compared to the conventional group [105 min (95% CI: 96–113)]. The aortic cross-clamp time was also reduced in the Perceval group [49 min (95% CI: 47–54)] versus the conventional group [56 min (95% CI: 51–62)]. Compared with the conventional valve, the Perceval group demonstrated a larger EOA [2.14 ± 0.48 cm^2^ vs. 1.55 ± 0.42 cm^2^], a lower mean transvalvular pressure gradient [10.5 ± 4.8 mmHg vs. 12.7 ± 7.3 mmHg], and a significantly lower incidence of PPM [3 patients (7%) vs. 27 patients (38%)]. The only notable difference in early postoperative events was a significantly lower incidence of transient low cardiac output requiring inotropic support in the Perceval group. Other in-hospital outcomes, including paravalvular leakage, new-onset atrial fibrillation, left bundle branch block, transient pacing, and permanent pacemaker implantation, were comparable between the groups. No significant differences were observed in the rates of acute kidney injury, stroke, or pulmonary complications. Two in-hospital deaths occurred in the conventional group, both attributed to right heart failure requiring extracorporeal life support in the context of a concomitant aortic root augmentation procedure.

### 3.3. Hemodynamic and Structural Outcomes Following AVR

As shown in Table 3, both subgroups demonstrated favorable reverse remodeling following AVR, with significant reductions in the IVST, PWT, LVESD, and LVEDD. These geometric changes were accompanied by reductions in the LVESV and LVEDV. Left ventricular mass regression was evidenced by a reduction in the indexed LVM upon follow-up echocardiographic surveillance in both groups. The valve hemodynamics improved similarly, with expanded indexed EOAs, reduced mean transvalvular pressure gradients, and lower velocity time integrals (VTIs), a flow-independent indicator of the aortic stenosis severity. Significant improvement in the left ventricular systolic function (fractional shortening and ejection fraction) was observed only in the Perceval group. Wall stress declined significantly in both subgroups, while E/e′, a marker of the LV filling pressure, remained unchanged. As shown in Table 3, postoperative comparisons revealed no significant differences in reverse remodeling or LV mass regression between the groups. However, the Perceval group showed significantly larger indexed EOAs, higher VTI ratios, lower mean gradients, and greater stroke volumes. The systolic pulmonary artery pressure also improved significantly in the Perceval group, while no between-group differences were found in the systolic function, wall stress, or filling pressure.

### 3.4. Late Adverse Events and Clinical Outcomes

**Figure 1:**
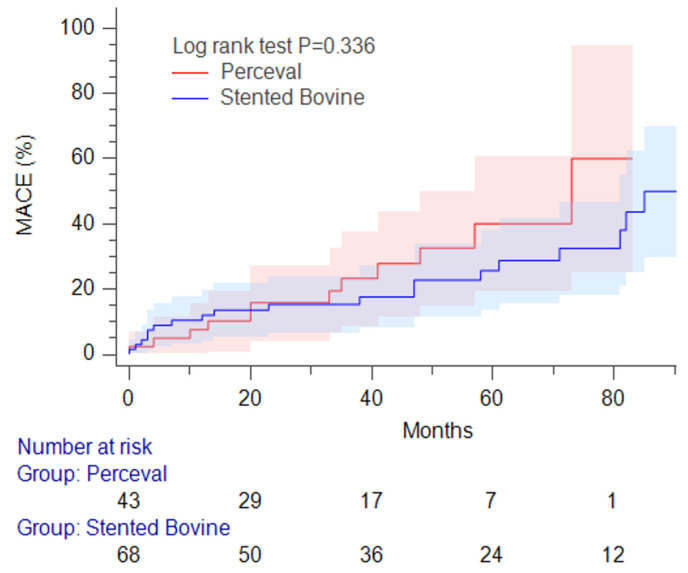
Kaplan–Meier analysis demonstrated no significant difference in the cumulative incidence of major adverse cardiovascular events between the Perceval and conventional valve groups (log-rank test, *p* = 0.336). The shaded areas represent the 95% confidence intervals for each survival curve.

As shown in Table 4, patient–prosthesis mismatch (PPM) was significantly more frequent in the conventional valve group, with 27 total cases, 19 of which were associated with the Mitroflow valve. Although the Perceval group had a numerically higher incidence of stroke, this difference did not reach statistical significance. A trend toward increased heart failure-related rehospitalization was observed in the conventional group, with 15 total events, including 11 among Mitroflow recipients. The prevalence of paravalvular leakage and late pacemaker implantation was comparable between the groups, as was the rate of redo AVR during follow-up. Table 5 displays the results of the Cox proportional hazards regression analysis for MACEs. Univariate analysis identified multiple predictors of MACEs, including the baseline E/e′, follow-up E/e′, systolic PAP, and indexed left ventricular mass (LVMI). However, in the adjusted multivariate analysis using stepwise selection, only the indexed LVMI emerged as a significant independent risk factor for MACEs. Kaplan–Meier analysis revealed no notable difference in the cumulative incidence of MACEs between the Perceval and conventional valve groups, as shown in Figure 1.

## 4. Discussion

In this retrospective analysis, the Perceval group exhibited larger EOAs, lower PPM rates, and reduced cardiopulmonary bypass and aortic cross-clamp durations compared to conventional valves. No significant differences were observed between groups in either the paravalvular leakage incidence or permanent pacemaker implantation requirements for conduction abnormalities.

### 4.1. Procedural Safety and In-Hospital Results

In terms of procedural safety, the Perceval group had no cases of valve extraction, aortic root bleeding, or laceration, and no in-hospital mortality. It also demonstrated significantly shorter cardiopulmonary bypass and aortic cross-clamp times, along with lower rates of transient low cardiac output syndrome requiring inotropic support. Although transient pacing and paravalvular leakage were slightly more frequent in the Perceval group, the differences were not statistically significant. The permanent pacemaker implantation rates were comparable between groups. Two in-hospital deaths occurred in the conventional group; both patients required aortic root enlargement for small annuli and developed left heart failure following prolonged bypass times.

### 4.2. Aortic Stenosis Pathophysiology and Prosthetic Evolution for AVR

Aortic stenosis is the most common structural valvular disease, affecting up to 25% of individuals over the age of 65, often in conjunction with hypertension and arteriosclerosis [1,2]. A stenotic aortic valve increases transvalvular pressure gradients and left ventricular (LV) afterload, elevating wall stress and impairing coronary flow reserve, which contributes to functional myocardial ischemia [32,33]. In response to chronic pressure overload, the LV undergoes concentric hypertrophy; however, this adaptation leads to diastolic dysfunction due to reduced vascularization and myocardial stiffening [7]. This cohort reflected key pathophysiologic features: 47% had bicuspid valves, 63% hypertension, 36% coronary artery disease, and 53% small annuli (≤21 mm). An indexed EOA < 0.65 cm^2^/m^2^ was seen in 88%, and 76% showed concentric hypertrophy, consistent with prior studies. The primary objective of AVR is to alleviate left ventricular outflow tract (LVOT) obstruction, decrease afterload, and restore optimal ventricular function. AVR enlarges the aortic orifice, reducing resistance to ejection and relieving pressure overload. This reduces left ventricular (LV) chamber pressure and dimensions, decreases the residual volume after systole, and lowers wall stress, which is proportional to end-systolic pressure and dimension according to the law of Laplace [9,26]. The resulting improvement in compliance enhances diastolic function and promotes more efficient ventricular filling [34]. The evolution of AVR has prioritized reducing the risk of PPM, primarily through surgical root enlargement techniques [12,13,14,15] and advancements in prosthetic valve design and material technology [16,28]. TAVI offers a less invasive alternative for high-risk aged patients, avoiding cardiopulmonary bypass and reducing ischemia–reperfusion injury [20,21,22]. However, its inability to decalcify the annulus may compromise prosthesis expansion, leading to higher rates of paravalvular leakage [23,24] and conduction disturbances due to oversizing or excessive radial force [30,35]. Additionally, a reduced EOA may impact the long-term durability [11]. Perceval valves provide a hybrid solution by combining surgical annular decalcification with sutureless anchoring, reducing operative times [18,28]. The self-expanding nitinol stent delivers radial force for secure, rapid deployment while minimizing cardiopulmonary bypass and cross-clamp durations. Its streamlined design improves flow and sealing, reducing paravalvular leakage. Optimal outcomes rely on precise sizing, accurate positioning, and avoiding deep implantation into the LV outflow tract to minimize conduction disturbances [19].

### 4.3. Reverse Remodeling and Mass Regression Following AVR

Both valve types were associated with favorable left ventricular reverse remodeling and mass regression. However, the Perceval group demonstrated significantly larger EOAs and a lower incidence of PPM. Reverse remodeling was reflected by reductions in the LVESD, LVEDD, and LV mass alongside decreased transvalvular gradients, contributing to lower wall stress [8,36,37]. Reductions in the end-systolic and end-diastolic volumes indicated decreased residual volume after ejection, suggesting more efficient ventricular emptying. Additionally, the leftward shift of the pressure–volume loop, along with a less steep end-diastolic pressure volume relationship (EDPVR) curve, reflected enhanced ventricular compliance, indicating improved diastolic function [37]. Together, these changes signify improved systolic performance and diastolic filling, consistent with favorable structural remodeling after AVR. The reversibility of extra-valvular cardiac damage (EVCD) significantly impacts the prognosis of aortic stenosis (AS) patients and may limit the long-term benefits of aortic valve replacement (AVR). Effective blood pressure control following AVR can enhance reverse remodeling by reducing left ventricular afterload [38].

### 4.4. Late Adverse Events and Identification of Risk Predictors

In the analysis of late adverse events, the Perceval group had a numerically higher rate of stroke, while heart failure-related rehospitalization and cardiac mortality were more frequent in the conventional group; however, none of these differences reached statistical significance. The rates of paravalvular leakage, permanent pacemaker implantation, and redo AVR for structural valve deterioration were comparable. Kaplan–Meier analysis revealed no significant difference in the cumulative incidence of MACEs between the groups. However, the adjusted Cox regression model identified the indexed left ventricular mass as an independent predictor of MACEs, suggesting that residual hypertrophy, despite partial regression following AVR, may contribute to the long-term risk prediction of MACEs and heart failure.

### 4.5. Clinical Implication

This study suggests that the Perceval valve may offer comparable clinical outcomes to those of conventional stented bioprostheses, with similar rates of paravalvular leakage and permanent pacemaker implantation. Both valve types showed equivalent long-term results, including heart failure-related rehospitalizations, and promoted reverse ventricular remodeling. However, the indexed LV mass independently predicted late adverse cardiovascular events, suggesting that residual left ventricular hypertrophy may persist despite hemodynamic improvement. Although the Perceval valve itself was not an independent predictor of outcomes in our analysis, its distinctive design and potential procedural benefits maintain its clinical relevance. Further large-scale studies with rigorous patient stratification are required to fully evaluate its long-term performance.

### 4.6. Study Limitations

This study has several limitations. First, it was conducted at a single institution with a relatively small sample size. Given the limitations of the sample size, there was an inherent risk of not fully accounting for potential biases in the multivariate analysis, which may lead to overfitting. To mitigate this risk, the Cox regression models were adjusted for key confounders, including age, sex, cardiovascular risk factors, including hypertension, systolic dysfunction, and a dilated LVEDD, despite the limited number of events. Second, longer follow-up is needed to fully assess structural valve deterioration, as this process can take several years to develop. The limited number of Perceval valve cases, combined with the fact that the Mitroflow valve accounted for most events in the conventional group, including PPM and heart failure-related rehospitalizations, may have influenced the comparative results. Third, echocardiographic assessments were not performed at standardized time intervals and were conducted by different operators, which may have introduced variability into the measurements of cardiac function and remodeling. Finally, as a retrospective and non-randomized study, this analysis is subject to potential selection bias and unmeasured confounding factors. While our findings demonstrate an association between hemodynamic and procedural outcomes, the observational design precludes definitive causal inference. Future prospective studies, ideally with randomized controlled trials, are needed to confirm these associations and investigate potential protective effects.

## 5. Conclusions

The Perceval valve is associated with hemodynamic and procedural benefits, particularly for small annuli and minimally invasive surgery, promoting favorable reverse remodeling and clinical outcomes comparable to those of conventional stented bioprostheses, supporting its use as an effective alternative in surgical aortic valve replacement.

## Figures and Tables

**Table 1:** Baseline characteristics of the 115 patients stratified by prosthesis type.

	Total N = 115	Perceval N = 44	Conventional N = 71	*p*
Age	65.0 ± 9.7	65.8 ± 7.6	64.5 ± 10.9	0.46
Male	67 (58)	28 (64)	39 (55)	0.44
Comorbidity				
Coronary artery disease	41 (36)	20 (45)	21 (30)	0.11
Hypertension	72 (63)	28 (64)	44 (62)	1.00
Diabetic	39 (34)	15 (34)	24 (34)	0.18
Dyslipidemia	85 (74)	34 (77)	51 (72)	0.66
Chronic renal disease	29 (25)	12 (27)	17 (24)	0.83
Hemodialysis	8 (7)	2 (5)	6 (8)	0.71
Cerebral infarct	9 (8)	3 (7)	6 (8)	1.00
Infective endocarditis	4 (3)	0 (0)	4 (6)	0.16
Atrial fibrillation	11 (10)	1 (2)	10 (14)	0.05
Left bundle branch block	17 (15)	5 (11)	12 (17)	0.59
Previous pacemaker implant	3 (3)	1 (2)	2 (3)	1.00
Bicuspid aortic valve	54 (47)	19 (43)	35 (49)	0.57
Aortic valve pathology				
Annulus diameter	21.3 ± 2.3	21.1 ± 2.1	21.4 ± 2.4	0.58
Annulus diameter ≤ 21 mm	61 (53)	23 (52)	38 (54)	1.00
Effective orifice area index ≥ 0.65, ≤0.85 cm^2^m^−2^	10 (9)	3 (7)	7 (10)	0.74
Effective orifice area index < 0.65 cm^2^m^−2^	101 (88)	39 (89)	62 (87)	1.00
Ventricular dysfunction				
LV ejection fraction < 55%	15 (13)	5 (11)	10 (14)	0.78
Systolic pulmonary artery pressure > 35 mm Hg	25 (22)	12 (27)	13 (18)	0.25
Remodel mode				
Concentric hypertrophy	87 (76)	33 (75)	54 (76)	1.00
Echocardiographic parameters				
Interventricular septum thickness, mm	13.8 ± 3.0	13.4 ± 2.9	14.1 ± 3.1	0.197
Posterior wall thickness, mm	12.8 ± 2.8	12.8 ± 2.7	12.8 ± 2.8	0.918
LV end-systolic dimension, mm	30.8 ± 7.7	31.4 ± 7.7	30.5 ± 7.8	0.509
LV end-diastolic dimension, mm	50.1 ± 7.4	50.9 ± 6.4	49.5 ± 8.0	0.336
LV end-systolic volume, mL	41.3 ± 27.9	43.0 ± 29.9	40.2 ± 26.7	0.596
LV end-diastolic volume, mL	122.3 ± 43.8	126.0 ± 37.6	119.9 ± 47.3	0.473
Indexed LV mass, g$\cdot m^{-2}$	166.1 ± 62.3	163.7 ± 66.9	167.5 ± 59.8	0.747
Indexed effective orifice area, ${cm}^{2}\cdot m^{-2}$	0.48 ± 0.19	0.48 ± 0.16	0.48 ± 0.21	0.958
Mean pressure gradient, mm Hg	44.8 ± 19.1	46.1 ± 20.1	43.9 ± 18.5	0.559
Velocity–time integral ratio, %	0.25 ± 0.09	0.25 ± 0.10	0.24 ± 0.09	0.743
Wall stress, Kdyne·cm^−2^	98.1 ± 35.1	102.4 ± 34.6	96.2 ± 41.3	0.411

Data are presented as number (%), mean ± SD.

**Table 2:** Operative characteristics and in-hospital adverse events.

	Total N = 115	Perceval N = 44	Conventional N = 71	*p*
Operative Characteristics				
Cardiopulmonary bypass time, min	100 (93 to 105)	93 (88 to102)	105 (96 to 113)	0.02
Aortic clamp time, min	53 (53 to 55)	49 (47 to 54)	56 (51 to 62)	0.02
Ministernotomy	88 (77)	37 (84)	51 (72)	0.18
Previous aortic valve replacement	6 (5)	1 (2)	5 (7)	1.00
Coronary artery bypass graft	18 (16)	6 (14)	12 (17)	0.79
Root augmentation	6 (5)	0 (0)	6 (8)	0.08
Pulmonary vein ablation	7 (6)	1 (2)	6 (8)	0.25
Hospital Outcomes				
Re-exploration for bleeding	4 (3)	1 (2)	3 (4)	1.00
Transient low cardiac output	23 (20)	3 (7)	20 (28)	<0.01
Intra-aortic ballon pump	1 (1)	0 (0)	1 (1)	1.00
Extracorporeal life support	3 (3)	1 (2)	2 (3)	1.00
Acute kidney injury	11 (10)	4 (10)	7 (10)	1.00
Renal replacement therapy	10 (9)	3 (7)	7 (10)	0.74
Cerebral infarct	2 (2)	1 (2)	1 (1)	1.00
Ventilator support > 48 hr	7 (6)	2 (5)	5 (7)	0.71
Pulmonary complication	9 (8)	2 (5)	7 (10)	0.48
Paravalvular leakage	4 (3)	3 (7)	1 (1)	0.16
New atrial fibrillation	18 (16)	5 (11)	13 (18)	0.43
New left bundle branch block	24 (21)	11 (25)	13 (18)	0.35
Transient pacing	22 (19)	11 (25)	11 (15)	0.23
Permanent pacemaker implantation	5 (4)	2 (5)	3 (4)	1.00
Death	2 (2)	0 (0)	2 (3)	0.52

Data are presented as number (%); in subgroup as number (% within a subgroup); median (95% CI).

**Table 3:** Changes in geometry, hemodynamics following AVR: baseline vs. follow-up by echocardiography.

	Total N = 115			Perceval N = 44			Conventional N = 71				
	Baseline	Follow-Up	*p*	Baseline	Follow-Up	*p*	Baseline	Follow-Up	*p*	* *p*	† *p*
Geometry											
IVST, mm	13.8 ± 3.0	12.3 ± 3.1	<0.01	13.4 ± 2.9	12.4 ± 3.1	0.09	14.1 ± 3.1	12.2 ± 3.1	<0.01	0.12	0.78
PWT, mm	12.8 ± 2.8	11.5 ± 2.4	<001	12.8 ± 2.7	11.6 ± 2.4	<0.01	12.8 ± 2.8	11.4 ± 2.3	<0.01	0.92	0.64
LVESD, mm	30.8 ± 7.7	27.1 ± 6.1	<0.01	31.4 ± 7.7	26.7 ± 5.8	<0.01	30.5 ± 7.8	27.4 ± 6.3	<0.01	0.51	0.58
LVEDD, mm	50.1 ± 7.4	45.5 ± 6.2	<0.01	50.9 ± 6.4	45.6 ± 6.0	<0.01	49.5 ± 8.0	45.5 ± 6.4	<0.01	0.34	0.93
LVESV, mL	41.3 ± 27.9	27.1 ± 20.8	<0.01	43.0 ± 29.9	27.8 ± 16.4	<0.01	40.2 ± 26.7	26.6 ± 23.2	<0.01	0.60	0.76
LVEDV, mL	122.3 ± 43.8	89.1 ± 41.3	<0.01	126.0 ± 37.6	95.8 ± 34.0	<0.01	119.9 ± 47.3	85.1 ± 44.9	<0.01	0.47	0.18
LVM, g	281.3 ± 108.6	187.49 ± 1.4	<0.01	282.8 ± 117.6	205.0 ± 80.1	<0.01	280.4 ± 103.5	176.7 ± 96.5	<0.01	0.91	0.11
Indexed LVM, g·m^−2^	166.1 ± 62.3	110.3 ± 51.6	<0.01	163.7 ± 66.9	118.1 ± 44.6	<0.01	167.5 ± 59.8	105.6 ± 55.2	<0.01	0.75	0.21
Hemodynamics											
EOA, cm^2^	0.81 ± 0.32	1.79 ± 0.53	<0.01	0.82 ± 0.24	2.14 ± 0.48	<0.01	0.81 ± 0.36	1.55 ± 0.42	<0.01	0.85	<0.01
Indexed EOA, cm^2^·m^−2^	0.48 ± 0.19	0.97 ± 0.39	<0.01	0.48 ± 0.16	1.2 ± 10.33	<0.01	0.48 ± 0.21	0.82 ± 0.35	<0.01	0.96	<0.01
MPG, mm Hg	44.81 ± 9.1	11.8 ± 6.5	<0.01	46.1 ± 20.1	10.5 ± 4.8	<0.01	43.9 ± 18.5	12.7 ± 7.3	<0.01	0.56	0.06
VTI ratio, %	0.25 ± 0.09	0.53 ± 0.16	<0.01	0.25 ± 0.10	0.61 ± 0.02	<0.01	0.24 ± 0.09	0.48 ± 0.13	<0.01	0.74	<0.01
Function											
SV, mL	81.6 ± 24.3	62.0 ± 27.2	<0.01	84.3 ± 22.7	68.0 ± 23.2	<0.01	79.92 ± 5.2	58.4 ± 28.9	<0.01	0.34	<0.01
FS, %	38.9 ± 8.4	40.7 ± 7.6	0.01	38.8 ± 8.7	41.7 ± 8.0	0.03	39.0 ± 8.3	40.1 ± 7.3	0.14	0.88	0.28
EF, %	68.2 ± 11.2	70.9 ± 9.7	<0.01	67.9 ± 11.9	71.9 ± 9.8	0.02	68.4 ± 10.8	70.1 ± 9.7	0.09	0.82	0.36
Wall stress, Kdyne·cm^−2^	98.1 ± 35.1	80.1 ± 31.3	<0.01	102.4 ± 34.6	78.3 ± 35.8	<0.01	96.2 ± 41.3	81.4 ± 28.0	0.03	0.41	0.62
E/e′ ratio	16.2 ± 8.3	14.7 ± 7.3	0.06	16.4 ± 6.7	15.0 ± 6.8	0.14	15.9 ± 9.6	14.4 ± 7.8	0.21	0.79	0.96
Systolic PAP, mm Hg	24.9 ± 10.5	24.0 ± 11.5	0.48	25.4 ± 11.0	21.8 ± 7.9	0.05	24.5 ± 10.2	25.5 ± 13.3	0.54	0.26	<0.01

Data are presented as number of patients (%) (n/N), mean ± SD. Abbreviations: IVST, interventricular septum thickness; PWT, posterior wall thickness; LVESD, left ventricular end-systolic dimension; LVEDD, left ventricular end-systolic dimension; LVESV, left ventricular end-systolic volume; LVEDV, left ventricular end-systolic volume; SV, stroke volume; FS, fraction shortening; EF, ejection fraction; LVM, left ventricular mass; EOA, effective orifice area; MPG, mean pressure gradient; VTI ratio, velocity–time integral ratio; E/e′: E, peak velocity of early mitral inflow; e′, early diastolic velocity of the mitral annular tissue doppler; Systolic PAP, systolic pulmonary artery pressure. * *p* indicates comparison between Perceval and Conventional at baseline; † *p* indicates comparison between Perceval and Conventional following AVR.

**Table 4:** Late adverse events following AVR.

	Total N = 115	Perceval N = 44	Conventional N = 71	*p*
Coronary-related intervention	8 (7)	3 (7)	5 (7)	1.00
Stroke	11 (10)	7 (16)	4 (6)	0.10
HF-related rehospitalization	20 (17)	5 (11)	15 (21)	0.21
Major cardio-cerebral events	31 (27)	12 (27)	19 (27)	1.00
Paravalvular leakage	6 (5)	3 (7)	3 (4)	0.67
Permanent pacemaker implantation	4 (3)	2 (5)	2 (3)	0.64
Patient–prosthesis mismatch	30 (26)	3 (7)	27 (38)	<0.01
Infective endocarditis	1 (1)	0 (0)	1 (1)	1.00
Redo AVR	6 (5)	2 (5)	4 (6)	1.00
Cardiac-related mortality	11 (10)	2 (5)	9 (13)	0.20
All-cause mortality	12 (11)	2 (5)	10 (14)	0.13

Data are presented as number (%), mean ± SD. Abbreviation: HF, heart failure; patient–prosthesis mismatch indicates ≤0.85 cm^2^m^−2^.

**Table 5:** Cox proportional hazard regression for MACEs following AVR.

	Univariate			Multivariate *		
	HR	95% CI	*p*	HR	95% CI	*p*
Preoperative						
E/e′ ratio ≥ 15	1.05	1.02 to 1.09	<0.01			
Systolic PAP ≥ 35 mm Hg	2.11	0.96 to 4.66	0.06			
Follow-up						
indexed LV mass	1.02	1.01 to 1.03	<0.01	1.02	1.01 to 1.03	<0.01
E/e′ ratio ≥ 15	1.08	1.03 to 1.12	<0.01			
Systolic PAP ≥ 35 mm Hg	2.59	1.26 to 5.31	<0.01			
PPI	1.98	0.47 to 8.36	0.36			
Paravalvular leakage	2.46	0.92 to 6.55	0.07			
Redo AVR	2.17	0.87 to 5.41	0.09			
Perceval valve	1.93	0.88 to 4.22	0.10			

Data are presented as median (95% CI). HR, hazard ratio. Abbreviations are depicted as in Table 3. * Models were adjusted for age, sex, cardiovascular risk factors (including hypertension, baseline LVEF < 50%, and LVEDD > 55 mm), using stepwise method.

## Data Availability

The data presented in this study are available upon request from the corresponding author. The data are not publicly available due to privacy and ethical reasons.

## Abbreviations

AS	Aortic Valve Stenosis
AVR	Aortic Valve Replacement
CABG	Coronary Artery Bypass Graft
CPB	Cardiopulmonary Bypass
EDD	End-Diastolic Dimension
EDV	End-Diastolic Volume
ESD	End-Systolic Dimension
ESV	End-Systolic Volume
EF	Ejection Fraction
FS	Fractional Shortening
IVST	Interventricular Septum Thickness
LVM	Left Ventricular Mass
LVMI	Indexed Left Ventricular Mass
MACE	Major Adverse Cardiac–Cerebral Event
PPI	Permanent Pacemaker Implantation
PPM	Patient–Prosthesis Mismatch
PVL	Paravalvular Leakage
RWT	Relative Wall Thickness
SPAP	Systolic Pulmonary Arterial Pressure
SV	Stroke Volume
TAVI	Transcatheter Aortic Valve Implantation
WS	Wall Stress
VIS	Vasoactive Inotropic Score
VTI	Velocity Time Integral
